# Prognostic significance of systemic pan-immune-inflammation value in locally advanced cervical cancer

**DOI:** 10.3389/fonc.2024.1492251

**Published:** 2024-10-28

**Authors:** Shu Yan, Xin Gong, Rui Liu, Xiaojing Jia

**Affiliations:** Department of Tumor Radiotherapy, The Second Hospital of Jilin University, Changchun, China

**Keywords:** pan immune inflammation value, locally advanced cervical cancer, prognostic prediction, concurrent chemoradiotherapy, immune-inflammatory biomarkers

## Abstract

**Objective:**

This study investigates the significance of systemic pan-immune inflammation value (PIV) prior to concurrent chemoradiotherapy (CCRT) in predicting the therapeutic efficacy as well as prognosis of patients with locally advanced cervical squamous cell carcinoma.

**Methods:**

A retrospective analysis was conducted on the clinical data of 847 patients with locally advanced cervical cancer (LACC) treated at the Second Hospital of Jilin University between 2016 and 2020. All patients underwent radical CCRT, including platinum-based sensitizing chemotherapy. The PIV was measured as given by: (platelet count × neutrophil count × monocyte count)/lymphocyte count. Logistic regression analysis was utilized to study the effect of PIV on therapeutic response in LACC patients and Kaplan–Meier survival together with Cox proportional hazard model to assess its impact on prognosis.

**Results:**

With the therapeutic effect as the endpoint, the optimal cutoff of PIV (356.0099) was signified via the receiver operating characteristics curve, and patients were grouped and compared based on this value. PIV was determined as an independent predictor of the therapeutic effect in CCRT for LACC (hazard ratio (HR) 1.696, 95% confidence interval (CI) 1.111–2.590). PIV was also an independent predictor of overall survival (OS) (HR 0.540, 95% CI 0.409–0.713, p<0.001) as well as disease-free survival (DFS) (HR 0.680, 95% CI 0.528–0.876, p=0.003). Compared to the low-PIV group, it was noted that individuals with a high PIV exhibited a poorer therapeutic effect and shorter OS and DFS.

**Conclusion:**

Patients with LACC and high PIV had poorer therapeutic outcomes and shorter OS and DFS. Our results may provide PIV as a new prognostic biomarker for LACC, if future prospective studies with large patient numbers support our findings.

## Introduction

Cervical cancer is globally recognized as the fourth most well-known death caused by cancers among women, presenting a substantial risk to their overall health and longevity across the globe (1). The conventional treatment protocol for locally advanced cervical cancer (LACC) typically includes a synergistic application of localized radiotherapy and systemic chemotherapy. However, the efficacy of such treatments often faces significant challenges, including issues, such as local relapse and the occurrence of distant metastasis (2–5). This necessitates the rational prediction of patient survival times to formulate more personalized treatment plans at an early stage, with such a wide range of factors affecting these predictions being layered (6, 7). Although the staging criteria defined by the International Federation of Gynecology and Obstetrics (FIGO) provide an important reference for clinical management in LACC, experience from practice suggests that patients with similar stages can have markedly different responses to treatment. This demonstrated that patient-specific factors play a vital role in determining the effectiveness of interventions (8).

The link between inflammation and cancer has been thoroughly explored in recent years to offer new insights into treating oncological diseases (9, 10). Previous clinical studies have indicated that chronic inflammation can induce malignant cell proliferation, promoting the formation of malignant tumors and affecting patient prognosis (11). Furthermore, the inflammatory microenvironment within tumors significantly impacts their response to anti-cancer therapies (12). A growing body of evidence indicates that certain specific immuno-inflammatory biomarkers (IIBs), such as neutrophil, lymphocyte, and monocyte levels, reflect the equilibrium of the host’s immunoinflammatory environment, these biomarkers are crucial for predicting cancer prognosis and are associated with carcinogenesis and tumor progression (13, 14). Additionally, the neutrophil-to-lymphocyte ratio (NLR), platelet-to-lymphocyte ratio (PLR), lymphocyte-to-monocyte ratio (LMR), and systemic inflammatory index have demonstrated significant predictive value in the clinical prognosis of a wide range of cancer types, especially in malignant tumors where patients exhibit chemoresistance (15–19). As a solid malignant tumor, cervical cancer prognosis is similarly influenced by these IIBs, prior research has indicated that certain hematological indicators including prognostic nutritional index (PNI), PLR, and LMR can serve as significant prognostic factors for cervical cancer outcomes (20). The Pan-Immune-Inflammation Value (PIV), first introduced in a 2020 study, is a calculated metric derived from four blood parameters: platelets, neutrophils, monocytes, and lymphocytes (21). This value reflects the balance between host immunity and inflammation, it serves as an accessible indicator for evaluating cancer outcomes and has been identified as an independent predictor of prognosis in metastatic colorectal cancer patients (22). It was also proven in different other malignancies like oral, esophageal, and head & neck tumors (23–26). Nevertheless, an exhaustive investigation of the correlation between the PIV and the clinical traits, along with its prognostic significance, in the context of LACC remains to be conducted.

Consequently, this research’s goal to explore the relationship the PIV with the clinical profile of patients diagnosed with LACC. Additionally, we sought to assess the prognostic predictive efficacy of PIV for CCRT in LACC, using both uni- and multivariate survival analyses. It is anticipated that our findings will yield novel theoretical insights and offer practical directives, thereby enhancing the precision and personalization of therapeutic approaches for LACC.

## Methods and materials

### Patients

In this research, we retrospectively examined the clinical records of 847 patients with LACC, all of whom received treatment at the Department of Radiotherapy in our hospital from 2016 to 2020. The inclusion criteria include: (1) diagnosis of cervical squamous cell carcinoma by histopathology, (2) staging as IB3-IVA according to the 2018 FIGO staging, and (3) serum laboratory results from our hospital’s automatic blood analyzer within 5 days prior to treatment. The exclusion criteria include: (1) the occurrence of other primary malignant tumors; (2) previous radiotherapy, chemotherapy, or radical surgery before treatment; (3) acute or chronic infections; (4) hematological or other autoimmune diseases; (5) incomplete clinical data; and (6) loss to follow-up. The flowchart of the recruitment process is shown in **Supplementary Figure S1**.

All patients provided informed consent and the study obtain approval from the Declaration of Helsinki. This study protocol was approved by the Ethics Committee of the Second Hospital of Jilin University (2024-030). The PIV was calculated using the formula: (neutrophil count [109/L] × platelet count [109/L] × monocyte count [109/L])/lymphocyte count [109/L] (22), derived from the results of the automatic blood analyzer within 5 days prior to treatment.

All patients with LACC were untreated prior to receiving CCRT. The clinical data collected included medical history, laboratory data, physical examination, imaging examinations (pelvic CT or MRI), bone scans, positron emission tomography-CT, as well as lymph node ultrasonography. Older adult patients were defined as those aged >65 years. The clinical characteristic parameters included in the study were patient age, comorbidities, gravidity and parity, lymph node metastasis status, histopathological results, tumor size before and after treatment, degree of change, parametrial invasion, and lymph node metastasis status. Given that patient recruitment started in 2016 and ended in 2020, all included patients’ FIGO staging was adjusted to the 2018 FIGO staging. All patients underwent radical CCRT (45–50.4 Gy) and concurrently received platinum-based sensitizing chemotherapy. The effectiveness of tumor therapy was gauged by the levels of sensitivity, categorized as complete and partial responses (CR&PR), and by the tolerance, which included stable and progressive diseases (SD&PD), according to the Response Evaluation Criteria in Solid Tumors (RECIST). These assessments were made by reviewing the patient outcomes during a 6-month post-treatment surveillance period. After treatment, a structured follow-up plan was initiated which required patients to be checked every three months within first year after which checking was to be relative with time in between six to twelve months intervals. Each follow-up entailed a review of the imaging materials and laboratory work done while still in our hospital. The final follow-up deadline was December 30, 2023, or the patient’s death. The primary endpoint of the study was 3-year OS of the patients.

### Data analysis

We employed the SPSS software (version 26; IBM Corp., Armonk, NY) for all statistical analyses. For descriptive statistics, categorical data are presented as numbers as well as percentages meanwhile continuous data are denoted as interquartile range (IQR) and median. PIV-associated optimal threshold was determined by the receiver operating characteristic (ROC) curve as well as Youden’s J statistic with tumor response as a binary outcome. Logistic regression analysis was used to assess the factors that predicted sensitivity of tumors to chemoradiotherapy based on OR and 95% CI. Kaplan-Meier plots were constructed for overall survival and tested using log rank test. OS and disease-free survival (DFS) were measured using the Cox proportional hazards model, adjusting for integer age at diagnosis; the hazard ratios (HRs), with their 95% CIs, are presented. The threshold for statistical significance was represented as a p-value of less than 0.05.

## Findings

### Patient characteristics

In this research, from a pool of 1,194 patients extracted from our hospital’s medical records spanning 2016 to 2020, a total of 847 patients diagnosed with LACC (classified according to FIGO 2018 as stages IB3-IVA) were selected based on the study’s eligibility criteria. The patients’ mean age at diagnosis was 55 (IQR, 49−62) years. Predominantly, the study enrolled patients with stage IIB LACC, constituting 55.4% of the study’s cohort. Each patient underwent pelvic irradiation along with chemotherapy regimens based on platinum, with 471 (55.6%) undergoing less than five cycles of chemotherapy, whereas 376 (44.4%) completed five or more cycles of concurrent chemotherapy. A total of 808 patients (95.4%) underwent brachytherapy. The total treatment time was 8 weeks (56 days) in 448 cases (52.9%) and <8 weeks in 399 cases (47.1%). Detailed clinical and pathological characteristics and baseline hematological indicators of the patients with LACC are shown in **Table 1**. The detailed distribution of PIV, platelets, lymphocytes, monocytes, and neutrophils is illustrated in **Supplementary Figure S2**.

**Table 1:** Patient characteristics (n=847).

Variable	Characteristics
**Median age (IQR)**	55 (49−62)
Complication	
**Hypertension**	110 (13%)
**Diabetes**	40 (4.7%)
**Cardiovascular disease**	38 (4.5%)
**Others**	16 (1.9%)
Tumor size	
**≤4 cm**	310 (36.6%)
**>4 cm**	537 (63.4%)
Staging (FIGO 2018)	
**IB3-IIA**	57 (6.7%)
**IIB**	469 (55.4%)
**IIIA-IIIB**	197 (23.3%)
**IIIC-IVA**	124 (14.6%)
**Parauterine**	779 (92%)
**Vagina**	48 (5.7%)
Lymph node (yes/no)	
**Pelvic lymph**	88 (10.4%)
**Inguinal lymph**	259 (30.6%)
**Paravascular iliac lymph**	387 (45.7%)
**Paraaortic lymph**	14 (1.7%)
**Supraclavicular lymph**	11 (1.3%)
**Brachytherapy**	808 (95.4%)
EQD2 (point A)	
**<80 Gy**	283 (33.4%)
**≥80 Gy**	564 (66.6%)
Chemotherapy cycle	
**<5th**	471 (55.6%)
**≥5th**	376 (44.4%)
Overall treatment time (d)	
**<56**	399 (47.1%)
**≥56**	448 (52.9%)
**Platelets (×10^3^ µL^−1^)**	255.00 (205.9−309.0)
**Lymphocyte (×10^3^ µL^−1^)**	1.50 (0.90−2.00)
**Neutrophil (×10^3^ µL^−1^)**	4.10 (2.88−5.40)
**Monocyte (×10^3^ µL^−1^)**	0.40 (2.88−5.40)
**Albumin (g/L)**	42.5 (40.20−44.45)
**HB (g/L)**	127.00 (113.00−136.00)
**NLR**	2.91 (2.00−4.48)
**PLR**	178.00 (131.50−270.00)
**LMR**	0.34 (0.22−0.50)
**PIV**	278.73 (163.53−489.19)

FIGO, International Federation of Gynecology and Obstetrics; EQD2, 2-Gy equivalent dose; HB, hemoglobin; NLR, neutrophil-to-lymphocyte ratio; PLR, platelet-to-lymphocyte ratio; LMR, lymphocyte-to-monocyte ratio; PIV, pan-immune-inflammation value.

### Comparison of PIV with other IIBs

**Figure 1:**
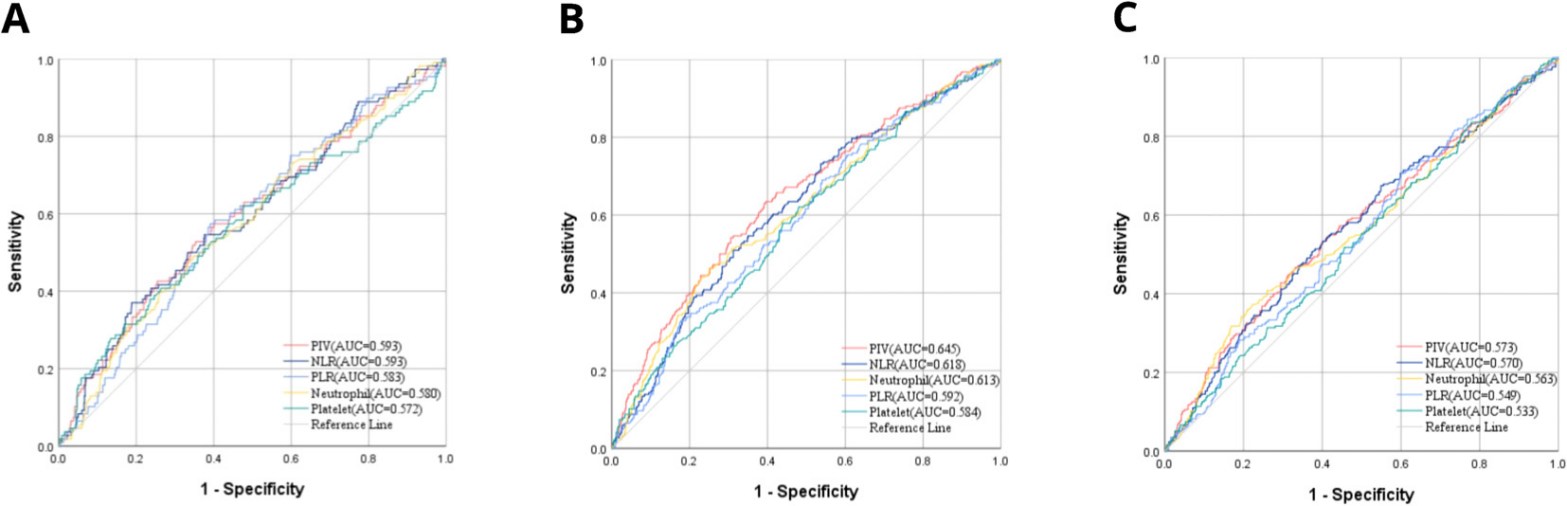
**(A)** AUC comparisons for CCRT responsiveness between PIV and other IIBs by ROC; **(B)** AUC comparisons for OS between PIV and other IIBs by ROC; **(C)** AUC comparisons for DFS between PIV and other IIBs by ROC.

To assess the efficacy of the pre-treatment PIV in predicting the responsiveness of patients with LACC to CCRT, we conducted a comparative analysis using the ROC curve. This analysis pitted PIV against traditional inflammatory markers, such as the PLR and NLR, as well as the individual components necessary for PIV computation, namely neutrophil and platelet counts. The outcomes of the ROC curve analysis indicated that PIV demonstrated superior predictive accuracy for the sensitivity of the patients to concurrent chemoradiotherapy (CCRT), with an area under the curve (AUC) of 0.593 (**Figure 1A**). This AUC was significantly higher than that of the PLR, NLR, neutrophil count and platelet count.

Moreover, the efficacy of these indicators in predicting OS and DFS was compared (**Figures 1B, C**). PIV was also superior to other comparative indicators in OS and DFS which were 0.645 and 0.543, accordingly. These results could have significant implications for the assessment of LACC patients’ sensitivity to CCRT in PIV.

### Relationship between PIV and patient characteristics

An ROC curve was generated Using tumor regression following LACC treatment as the endpoint, with PIV as the test variable. The coordinate points representing sensitivity and 1-specificity on the ROC curve were identified. Utilizing the formula: Youden index (J) = sensitivity + specificity - 1, we calculated the Youden index (J) for each coordinate point. The coordinate point corresponding to the maximum value of the Youden index (J) represents the optimal cutoff point for PIV. Using this aforesaid threshold value of 356.0099, patients were then classified into high- and low-PIV groups **Table 2** presents the association between clinical characteristics and PIV-based classification. Notable differences were observed between the high-PIV (PIV ≥356) and low-PIV (PIV <356) groups in terms of tumor dimensions (p less than 0.001), FIGO stage classification (p less than 0.001), parametrial invasion (p = 0.027), pelvic lymph node metastasis (p = 0.003), lower vaginal wall invasion (p = 0.020), para-aortic lymph node involvement (p = 0.027), and pre-treatment serum albumin levels (p < 0.001).

**Table 2:** Baseline clinical characteristics according to the PIV.

Variable	Low-PIV (n=529)	High-PIV (n=318)	P-Value
Age, years			
<65	437 (82.6%)	276 (86.8%)	0.120
≥65	92 (17.4%)	42 (13.2%)	
Complication (yes, no)			
Hypertension	74 (14%)/455 (86%)	36 (11.3%)/282 (88.7%)	0.292
Diabetes	29 (5.5%)/500 (94.5%)	11 (3.5%)/307 (96.5%)	0.241
Cardiovascular disease	25 (4.7%)/504 (95.3%)	13 (4.1%)/305 (95.9%)	0.734
Others	11 (2.1%)/518 (97.9%)	5 (1.6%)/313 (98.4%)	0.796
Diameter			
≤4 cm	231 (43.7%)	79 (24.8%)	<0.001
>4 cm	298 (56.3%)	239 (75.2%)	
FIGO			
IB3-IIA	44 (8.3%)	13 (4.1%)	<0.001
IIB	317 (59.9%)	152 (47.8%)	
IIIA-IIIB	108 (20.4%)	89 (28.0%)	
IIIC-IVA	60 (11.3%)	64 (20.144%)	
**Parauterine**	478 (90.4%)/51 (9.6%)	301 (94.7%)/17 (5.3%)	0.027
**Vagina**	22 (4.2%)/507 (95.8%)	26 (8.2%)/292 (91.8%)	0.020
Lymph node (yes/no)			
Pelvic lymph	42 (7.9%)/487 (92.1%)	46 (14.5%)/272 (85.5%)	0.003
Inguinal lymph	156 (29.5%)/373 (70.5%)	103 (32.4%)/215 (67.6%)	0.397
Paravascular iliac lymph	226 (42.7%)/303 (57.3%)	161 (50.6%)/157 (49.4%)	0.027
Para-aortic lymph	8 (1.5%)/521 (98.5%)	6 (1.9%)/312 (98.1%)	0.782
Supraclavicular lymph	6 (1.1%)/523 (98.9%)	5 (1.6%)/313 (98.4%)	0.755
**Albumin (g/L)**	42.90 (40.90−44.70)	41.70 (39.18−43.83)	<0.001
**HB (g/L)**	129.00 (119.00−137.00)	122.00 (103.00−132.25)	<0.001

FIGO, International Federation of Gynecology and Obstetrics; HB, haemoglobin.

### Predictors of therapeutic effect

PIV was closely related to the therapeutic effect, with fewer patients in the high-PIV group being more sensitive to treatment than those in the low-PIV group (90.34% vs. 82.08%, p=0.001). Following the outcomes of uni- and multivariate logistic regression analyses, PIV was an independent predictor of the therapeutic effect of CCRT for LACC (HR 1.696, 95% CI 1.111–2.590, p=0.014). In addition, FIGO staging, para-aortic lymph node metastasis, and total treatment time were independent predictors of therapeutic effects (**Table 3**).

**Table 3:** Logistic regression analyses for clinical characteristics.

Variable	Univariate analysis		Multivariate analysis	
	OR (95% CI)	P-value	OR (95% CI0	P-value
Age				
**(years, ≥65 vs.<65)**	0.993 (0.571−1.729)	0.981		
Complication				
**Hypertension**	1.193 (0.673−2.117)	0.546		
**Diabetes**	1.220 (0.500−2.977)	0.663		
**Cardiovascular disease**	0.575 (0.174−1.902)	0.364		
**Others**	0.977 (0.219−4.360)	0.976		
**Diameter**	1.297 (0.842−2.000)	0.238		
FIGO (2018)				
**IB3-IIA**	Reference		Reference	
**IIB**	11.250 (2.604−48.597)	0.001	7.796 (1.634−37.198)	0.010
**IIIA-IIIB**	3.855 (2.350−6.322)	<0.001	3.017 (1.466−6.206)	0.003
**IIIC-IVA**	2.815 (1.590−4.983)	<0.001	2.544 (1.191−5.473)	0.016
**Para-uterine**	1.557 (0.657−3.692)	0.315		
**Vagina**	1.632 (0.767−3.471)	0.204		
Lymph node (yes/no)				
**Pelvic lymph**	3.232 (1.929−5.416)	0.000	1.093 (0.501−2.383)	0.823
**Inguinal lymph**	1.397 (0.917−2.128)	0.120		
**Paravascular iliac lymph**	1.873 (1.243−2.824)	0.003	1.577 (1.030−2.413)	0.036
**Para-aortic lymph**	1.891 (0.519−6.889)	0.334		
**Supraclavicular lymph**	1.530 (0.326−7.179)	0.589		
**Brachytherapy**	1.827 (0.817−4.086)	0.142		
**EQD2**	0.760 (0.502−1.153)	0.197		
**Chemotherapy**	1.046 (0.697−1.570)	0.827		
**Time**	1.681 (1.106−2.533)	0.015	1.555 (1.010−2.389)	0.045
**PIV (high vs. low)**	2.047 (1.363−3.074)	0.001	1.696 (1.111−2.590)	0.014

FIGO, International Federation of Gynecology and Obstetrics; EQD2, 2-Gy equivalent dose; PIV, Pan-immune-inflammation value.

### Predictors of OS and DFS

**Figure 2:**
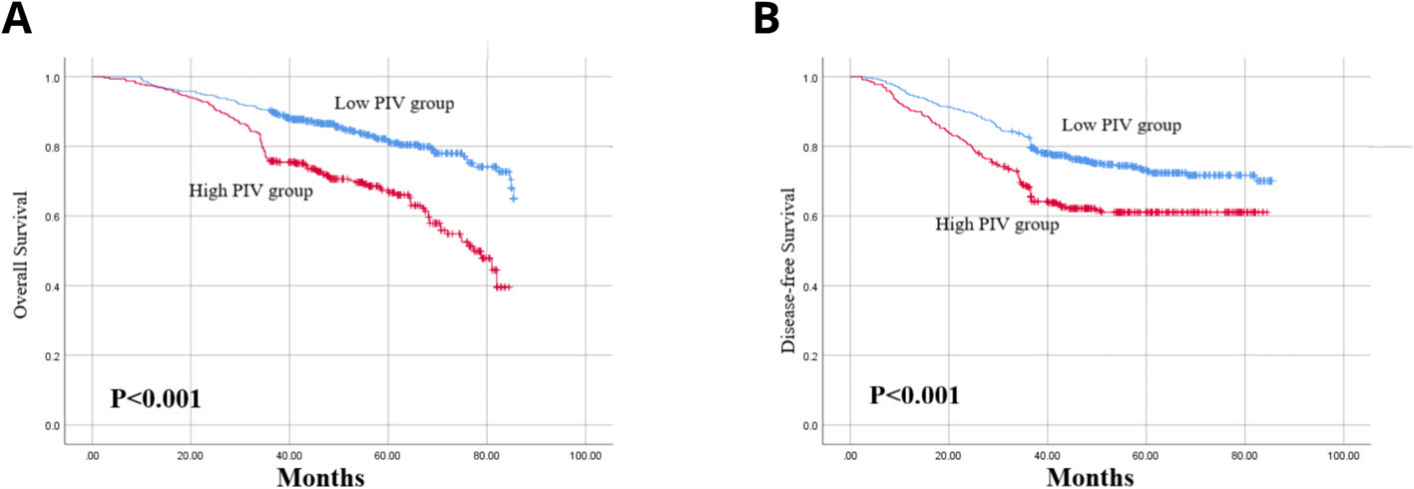
**(A)** Kaplan–Meier curves regarding OS; **(B)** Kaplan–Meier curves regarding DFS.

Employing the Kaplan-Meier method for survival analysis, we scrutinized the variance in OS and DFS among patients with LACC, distinguishing between the high and low PIV cohorts. Utilizing a critical PIV value of 356 as the threshold, the findings indicated that patients with elevated PIV scores notably experienced reduced OS and DFS in comparison to those with lower PIV scores, with the difference being statistically significant (p less than 0.001). This difference was confirmed via the log-rank test (**Figure 2**).

Furthermore, uni- and multivariate Cox proportional hazards models were utilised to analyse the data (**Tables 4**, **5**). Following the preliminary screening of variables via univariate analysis, a multivariate analysis was conducted. The data analysis demonstrated a strong and statistically significant association between the PIV score and both OS (HR 0.540, 95% CI 0.409–0.713, p<0.001) and DFS (HR 0.680, 95% CI 0.528–0.876, p=0.003). The research outcomes highlight that, in contrast to the group with lower PIV levels, the high-PIV group exhibited a respective 54% and 68% escalation in the risks of mortality and disease advancement. Consequently, PIV can be deemed an independent predictor of DFS as well as OS in patients with LACC.

**Table 4:** Univariate analysis.

Variable	OS		DFS	
	OR (95% CI)	P-value	OR (95% CI)	P-value
Age				
(years, ≥65 vs.<65)	0.945 (0.652−1.370)	0.765	0.993 (0.981−1.006)	0.280
**Diameter**	0.532 (0.388−0.728)	<0.001	0.611 (0.464−0.803)	<0.001
FIGO (2018)				
IB3	Reference		Reference	
II	0.182 (0.078−0.424)	<0.001	0.370 (0.188−0.731)	0.004
III	0.316 (0.224−0.446)	<0.001	0.557 (0.600−0.774)	0.001
IVA	0.591 (0.413−0.848)	0.004	0.892 (0.622−1.279)	0.532
**Parauterine**	0.442 (0.218−0.896)	0.023	0.632 (0.369−1.084)	0.096
**Vagina**	0.554 (0.353−0.869)	0.010	0.730 (0.452−1.179)	0.198
Lymph node (yes/no)				
Pelvic lymph	0.526 (0.361−0.768)	0.001	0.841 (0.571−1.238)	0.381
Inguinal lymph	0.751 (0.569−0.992)	0.043	0.791 (0.612−1.024)	0.075
Paravascular iliac lymph	0.764 (0.585−0.997)	0.048	0.879 (0.687−1.124)	0.303
Para-aortic lymph	1.121 (0.416−3.019)	0.821	1.148 (0.428−3.084)	0.784
Supraclavicular lymph	0.722 (0.268−1.944)	0.520	1.076 (0.345−3.360)	0.899
**Brachytherapy**	1.320 (0.753−2.314)	0.332	1.018 (0.570−1.817)	0.953
**EQD2**	1.341 (1.018−1.766)	0.037	1.135 (0.878−1.466)	0.334
**Chemotherapy**	1.136 (0.866−1.489)	0.356	1.156 (0.901−1.483)	0.253
**Time**	0.691 (0.525−0.909)	0.008	0.772 (0.602−0.990)	0.041
**PIV (high vs. low)**	0.439 (0.335−0.575)	<0.001	0.598 (0.467−0.765)	<0.001

FIGO, International Federation of Gynecology and Obstetrics; EQD2, 2-Gy equivalent dose; PIV, Pan-immune-inflammation value.

**Table 5:** Multivariate analysis.

Variable	OS		DFS	
	OR (95% CI)	P-value	OR (95% CI)	P-value
**Diameter**	0.697 (0.503−0.966)	0.030	0.731 (0.550−0.971)	0.031
FIGO (2018)				
**IB3**	Reference		Reference	
**II**	0.276 (0.117−0.653)	0.003	0.465 (0.234−0.926)	0.029
**III**	0.404 (0.284−0.575)	<0.001	0.634 (0.453−0.886)	0.008
**IVA**	0.633 (0.441–0.910)	0.014	0.909 (0.633−1.305)	0.604
**EQD2**	1.274 (0.963−1.685)	0.090		
**Time**	0.730 (0.553−0.963)	0.026		
**PIV (high vs. low)**	0.540 (0.409−0.713)	<0.001	0.680 (0.528−0.876)	0.003

FIGO, International Federation of Gynecology and Obstetrics; EQD2, 2-Gy equivalent dose; PIV, Pan-immune-inflammation value.

In the present research, tumor size and stage were established as significant, independent prognostic factors for OS as well as DFS in patients with LACC. Furthermore, 2-Gy equivalent dose (EQD2) and the cumulative duration of therapy were validated as independent predictors specifically for OS. These results highlight the importance of PIV in informing clinical management plans and predicting the prognosis with respect to patients with LACC.

## Discussion

In this research, we evaluated the predictive value of the PIV for therapeutic findings and prognosis in patients having LACC before initiating CCRT. By applying Cox proportional hazards model and Kaplan–Meier survival analysis, we identified PIV as a robust and independent prognostic factor significantly correlated with patient therapeutic outcomes and survival prognosis. High PIV often implies a poor prognosis. This is, to our knowledge the first study where we have demonstrated that PIV has a particular significance in patients with LACC.

Our findings indicate that elevated PIV serves not only as a reliable predictor of poor OS and DFS, but also as a significant independent marker for unfavorable treatment responses in patients with LACC. Compared to traditional indicators such as neutrophil counts, platelet levels, NLR, and PLR, PIV demonstrates superior predictive capability. Furthermore, its calculation relies on readily obtainable and cost-effective clinical parameters, establishing it as a prognostic indicator of considerable clinical relevance. The correlation between PIV and adverse treatment outcomes may be attributed to the immunosuppressive and pro-inflammatory states associated with elevated PIV levels. Such conditions could potentially facilitate tumor evasion, progression, and resistance to therapeutic interventions (27). An immunosuppressed milieu characterized by heightened neutrophil counts coupled with diminished lymphocyte levels may contribute to more aggressive tumor growth and metastasis (28, 29). Additionally, the pro-inflammatory state may enhance angiogenesis, invasion, and metastasis—factors indicative of poor cancer prognosis (30). Our results further underscore the necessity of integrating PIV alongside conventional clinical and pathological factors when forecasting treatment outcomes in LACC cases. Incorporating PIV into predictive models can augment prediction accuracy while informing the development of personalized therapeutic strategies.

The relationship between inflammation and the progression of cancer, as well as its correlation with an unfavorable prognosis, has been widely reported (23–26). In LACC treatment, the immune-inflammatory response is a critical prognostic factor that should not be overlooked. Previous studies have reported the significance of various immune-inflammatory cells, which include neutrophils, thrombocytes, lymphocytes, and monocytes, in predicting cancer prognosis (31). Subsequently, ratios involving immune cells have been widely proposed and used for cancer prognosis prediction, including the LMR, NLR, PLR, and other predictive factors (32). Based on these studies, PIV, a novel indicator, has been used in several disease prediction studies. Several studies have demonstrated that PIV is linked to the prognosis of various solid tumors, including oral malignant tumors, head and neck tumors, and other malignant tumors. Although the design of these studies varies, the potential of PIV as a predictive tool has gradually emerged, and its predictive value has been widely recognized.

Currently, there is a scarcity of studies examining the prognostic significance with respect to the PIV in cervical cancer. Nevertheless, previous studies indicated an increased PLR and NLR are related to worse survival in patients having cervical cancer (16, 33–37). Similarly, a decrease in the LMR and PNI is also linked to poorer clinical outcomes (38–40). Consistent with these results, the present research compared the significance of PIV with several conventional inflammatory indicators, including PLR, NLR, neutrophil count, and platelet count, to predict the therapeutic efficacy using the ROC curve and AUC. While PIV demonstrated an equivalent AUC to NLR in predicting treatment efficacy, it exhibited superior predictive performance for OS and DFS, underscoring its robust combined predictive capability. Furthermore, PIV emerged not only as an independent prognostic factor for LACC but also significantly correlated with poor therapeutic effects and shorter DFS and OS in patients with higher PIV.

It has been demonstrated that an increased neutrophil and platelet count and a decreased lymphocyte count is related to poorer prognoses of cancer patients in previously published studies (41–43). Therefore, as a ratio combining multiple immune factors, it is predictable that a high PIV is linked to a decreased OS. Changes in PIV not only provide comprehensive information about the tumor immune microenvironment but also reveal the immune status and body function, especially in its interaction with the tumor. Specifically, the PIV may indicate the intensity and efficiency of the patient’s immune surveillance. A higher PIV might reflect an immunosuppressive or inflammatory state, which is associated with tumor evasion and progression. In contrast, a lower PIV may reflect how well the antitumor immune response is working.

It is furthermore known that PIV has a close connection with tumor immunotherapy. The increased PIV change may reflect an intricate balance of inflammation and immunity responsible for determining the best tumor treatment options, prognosis prediction, and therapeutic response. Recently, studies have also identified PIV as an important prognostic factor in patients with recurrent or metastatic squamous cell carcinoma of the head and neck when treated with an immune checkpoint inhibitor. A multivariable PIV-based prognostic model incorporating Programmed Death Ligand 1 may provide a useful tool for future risk stratification and prognosis assessment (44). In addition, the studies showed a higher PIV correlated positively with worse survival among cancer patients post-immunotherapy treatment (45). This suggests that PIV is not just highly correlated with cancer progression, but can also influence the response of cancers to treatment include chemotherapy, radiotherapy and immunotherapy. Therefore, PIV may serve as a piece of creditable evidence for patient survival prognosis and disease progression detection.

In addition to the preliminary evidence of this study with respect to PIV in CCRT for LACC, some limitations are to be acknowledged. First and foremost, since it was a single-center, retrospective study the findings may not be generalizable. Secondly, all patients received platinum-based chemo- and radiotherapy, but potential heterogeneity with respect to the specific treatment plan may have impacted the results of this study. Moreover, the hospital adjuvant therapy could be another factor to affect the survival outcome. Third, despite the quite strict criteria for inclusion and exclusion in our study, confounding factors cannot be completely removed since all indicators were derived from peripheral blood samples of patients. Fourth, only patients with inflammation and hematological diseases or abnormal liver and kidney function or autoimmune diseases were excluded during data collection. However, individual differences in human beings are complex. This may explain why there is some variation among the study results. In the end, as this study was retrospective, data collection mainly focused on baseline characteristics, treatment responses, and prognostic data of patients. Detailed recording of side effects, toxic reactions, and other inflammatory markers during treatment was not conducted. Additionally, the follow-up time was relatively short in this study and additional surveillance for therapeutic efficacy-indexes during initial treatment improvement needs to be verified by longer duration of survival.

This study has some limitations, however it does suggest that PIV may be a promising candidate for evaluation of prognosis prior to CCRT in LACC patients. Future studies must be validated in a greater multi-center cohort to confirm the prognostic value of PIV, and are needed for exploring its potential applications as part of personalized medical strategies. Moreover, an in-depth study of the molecular biological mechanism underlying the interaction between PIV and CCRT is likely to establish more reliable treatment approaches. In addition, examination of the joint effects between PIV and these traditional biomarkers might help improve prognosis more accurately. Overall, the present study emphasizes the potential application of PIV in the prognostic assessment of LACC and highlights a new direction for future clinical practice and scientific research.

## Conclusion

We highlight the prognostic significance of PIV for patients with LACC. Our findings demonstrate that elevated PIV is related with unfavorable disease progression in LACC patients and identify PIV as an independent predictor of OS and DFS. The strategic application of PIV could improve the predictive accuracy of treatment responses and post-treatment survival durations in cervical cancer patients. We recommend that future studies should further validate the predictive value of PIV through prospective clinical trials and explore its potential application in personalized medicine. At the same time, we advocate for more in-depth mechanism studies of the interaction between PIV and the tumor microenvironment, as well as long-term follow-up studies to assess the impact of PIV on long-term survival and recurrence risk. Through these efforts, we expect to increase the clinical value of PIV as a prognostic tool, resulting in substantial improvements in treatment strategies and survival outcomes for patients with LACC.

## Data Availability

The raw data supporting the conclusions of this article will be made available by the authors, without undue reservation.

## Supplementary material

Supplementary Figure 1 Flowchart of recruitment process.

Supplementary Figure 2 Distribution of PIV, Platelet, Lymphocyte, Monocyte, and Neutrophil. (PLT, Platelet; LYM, Lymphocyte; MON, Monocyte; NEU, Neutrophil).
