# Supplementary figures and images for: Prognostic significance of systemic pan-immune-inflammation value in locally advanced cervical cancer
Source: Front Oncol. 2024 Oct 28;14:1492251. doi: 10.3389/fonc.2024.1492251 (PMC11551031; doi:10.3389/fonc.2024.1492251)

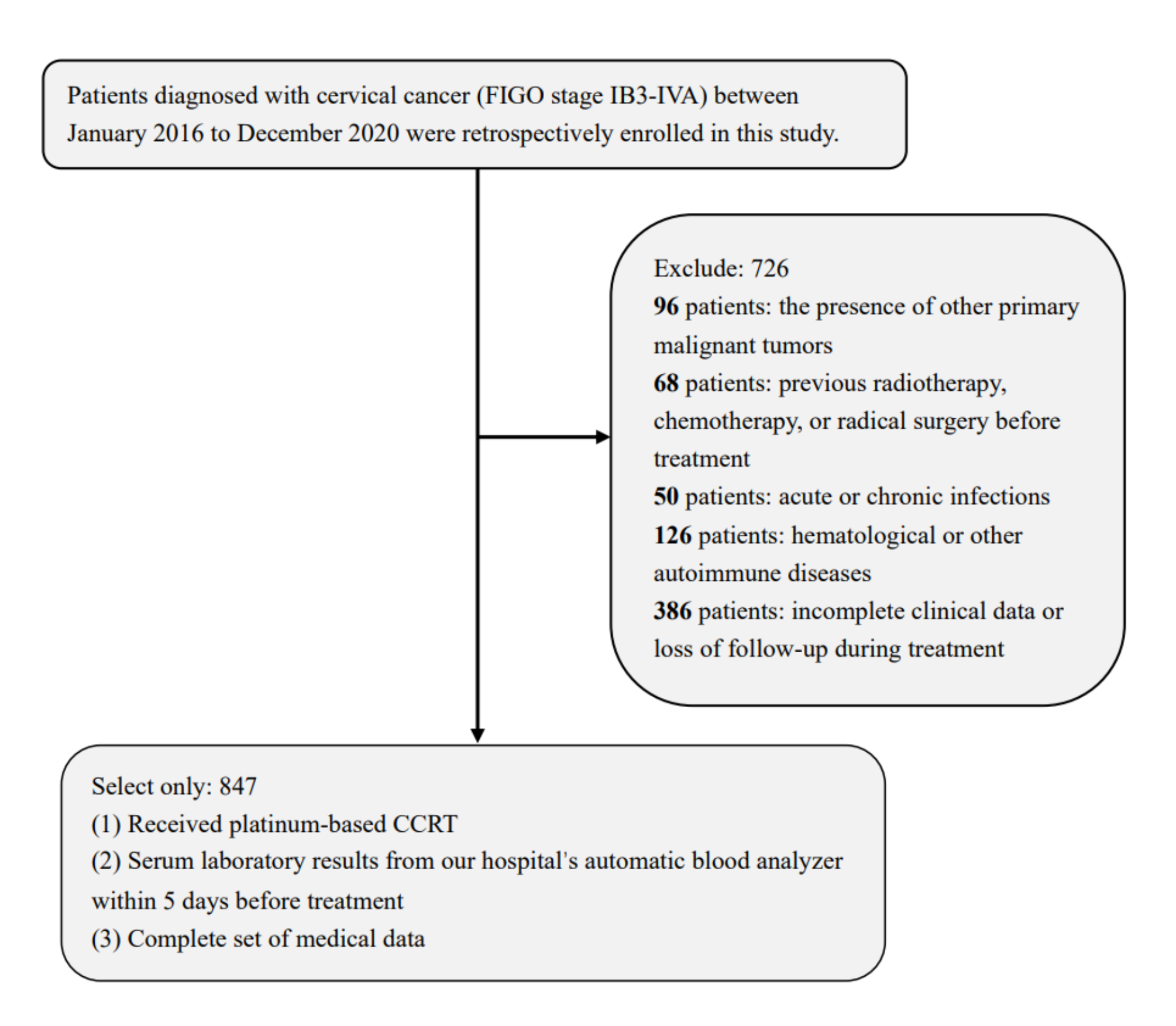

Supplement: Supplementary Figure 1 — Flowchart of recruitment process. [file Image1.tiff]

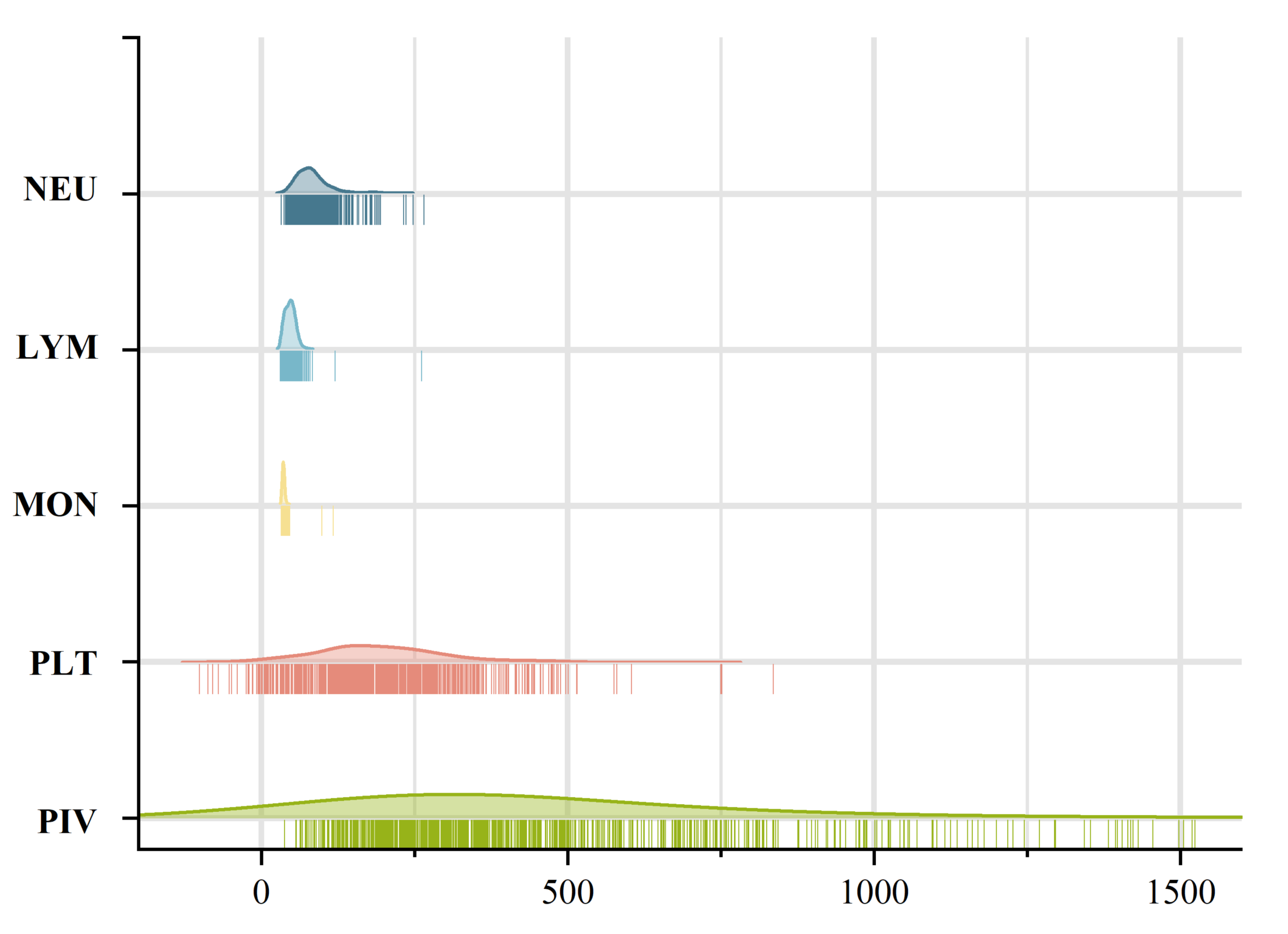

Supplement: Supplementary Figure 2 — Distribution of PIV, Platelet, Lymphocyte, Monocyte, and Neutrophil. (PLT, Platelet; LYM, Lymphocyte; MON, Monocyte; NEU, Neutrophil). [file Image2.tiff]
